# Interaction among general practitioners age and patient load in the prediction of job strain, decision latitude and perception of job demands. A Cross-sectional study

**DOI:** 10.1186/1471-2458-4-59

**Published:** 2004-12-07

**Authors:** Giedrius Vanagas, Susanna Bihari-Axelsson

**Affiliations:** 1 Kaunas University of Medicine, dept. Preventive Medicine, Kaunas, Lithuania

## Abstract

**Background:**

It is widely recognized and accepted that job strain adversely impacts the workforce. Individual responses to stressful situations can vary greatly and it has been shown that certain people are more likely to experience high levels of stress in their job than others. Studies highlighted that there can be age differences in job strain perception.

**Methods:**

Cross-sectional postal survey of 300 Lithuanian general practitioners. Psychosocial stress was investigated with a questionnaire based on the Reeder scale. Job demands were investigated with the Karasek scale. The analysis included descriptive statistics; logistic regression beta coefficients to find out predictors and interactions between characteristics and predictors.

**Results:**

Response rate was 66% (N = 197). Logistic regression as significant predictors for job strain assigned – duration of work in primary care; for job demands- age and duration of working in primary care; for decision latitude- age and patient load.

The interactions with regard to job strain showed that GP's age and job strain are negatively associated to a low patient load. Lower decision latitude for older GP age is strongly related to higher patient load. Job demands and GP age are slightly positively related at low patient load.

**Conclusions:**

Lithuanian GP's have high patient load and are at risk of stress, they have high job demands and low decision latitude. Older GP's perceive less strain, lower job demands and higher decision latitude in case of low patient load. Young GP's decision latitude has week association to patient load. Regarding to the changes in patient load younger GP's perceive it more sensitively as changes in job demands.

## Background

The issue of job stress is of utmost important to the public health community and working people because it adversely impacts the workforce. Strain has been considered as an environmental condition, as an appraisal of an environmental condition, as a response to an environmental condition, and as a form of relationship between environmental demands and a person's abilities to meet these demands. Although there are a lot of controversies about the epistemology of job strain, there is an agreement about it as a complex phenomenon related to health. In considering workplace-related stress, it should be recognized that stressors may occur because of individual characteristics of the worker as well as the work environment [1-5].

In general, physicians are at risk of stress. The main experienced pressures at work were uncertainty and insecurity, isolation, poor relationships with other doctors, disillusion with the role of the general practitioner and awareness of changing demands [6,7]. It has been demonstrated that negative feelings of tension, lack of time, excessive paper work among physicians take turnover to quality of care and was associated with poor clinical performance and patient's dissatisfaction [8-10].

The importance of job strain understanding as a problem for the general practitioners (GP's) was yielded by Appleton [11] in a study among 406 GP's. There was found that the prevalence of stress was 52%. Other studies also showed, that general practice is one of the most stressful workplaces among health care workers [12-15]. The specific characteristics that make general practice stressful are largely unknown. Sociodemographic factors such as age were depicted as independent predictors of vulnerability to GP's [16-21].

The personal and social conditions have influences on the relationship between age and stress. Continuing problems at work and job strain mostly affects young GP's [20,22]. On the contrary some studies showed that as a result of the age interaction, the total effects on job strain are twice larger in the sample of old persons as in the sample of young persons [21] and the age impact on job strain increases in successively in older age groups until retirement age [23]. The results of different studies showed that age also attribute to stress, anxiety, job satisfaction and quality of life for GP's [22-24]. It is shown that GP age and patient load have additive effects and increase vulnerability to stress [25] but still unknown how it interact with decision latitude and perception of job demands in general practice?

The aim of this study was to investigate physician's age, duration of work in primary care and patient load interactions with job strain, decision latitude and perception of job demands.

## Methods

### Target group

Lithuanian GP's.

### Study design

Cross – sectional study. A mailed survey of random national samples. Computerized random sampling was performed from the registry of Lithuanian physicians. The data collected through the questionnaires filled-in by the GP's.

### Sample size

Total number of GP's in Lithuania at the time was 1007 GP's. Sample size was calculated using EpiInfo 2000 Statcalc software which argued the sample size of 192 GP's with the 95% confidence level. From the previous studies the expected response rate was 63%. Therefore, it was decided to send questionnaires to 300 Lithuanian GP's. Our observed response rate was 66%. We collected 197 filled-in questionnaires.

### Assessment of Psychosocial Stress

Psychosocial stress in this study was investigated by a questionnaire based on the Reeder scale [26,27]. The Reeder scale uses four statements experienced in everyday stressful situations as "usually tense or nervous", "daily activities are extremely trying and stressful". The respondents should indicate whether each of the statements describe them. Each question has four alternative responses, which were coded using Likert-like scale.

A simple inversion of the Coulson scoring system (table 1) was used, giving a score of between 0 and 8 [28]. We have previously found analyses based on the Coulson approach to give very similar results to analyses based on the simple summation of scores [29].

**Table 1:** Coulson scoring system

**Score**	**Description**
0	No response on one or more statements.
1	Not at all' for all four statements.
2	'Not at all' for any three statements with any other response on the fourth.
3	'Not at all' for any two statements with 'Not very accurately' for the other two.
4	'Not at all' for any one or two statements with any other responses for the remainder but not those for a score of 3.
5	All other response sets not specified under 0, 1, 2, 3, 4, 6, 7, or 8.
6	'To some extent' to all four statements, or 'To some extent' for three statements with 'Exactly' for the fourth.
7	'Exactly' for any three statements with 'To some extent' or 'Not very accurately' for the fourth. Or 'Exactly' for two statements with 'To some extent' for two.
8	'Exactly' in response to all statements.

### Assessment of stressful work characteristics

Work characteristics were measured by the Karasek's Job Content Questionnaire. This instrument has two scales that measure stressful job character – job decision latitude and psychological workload demands. This model, also known as the "job strain" model [30-32].

Psychological workload demands were defined by questions such as "working very fast," "working very hard," "doing so many things".

Job decisions latitude was measured within questions as: "always must learn for new skills", "working a lot".

A four point Likert – like scale was used with the coding from 4 to 1 for series, so that the responses were summarised to give a score [33].

### Statistical analysis

Data were computed, coded and analyzed using Statistical Package for the Social Sciences for Windows version 11.0 (SPSS Inc) and Microsoft Excel 2000. The analysis included descriptive statistics; logistic regression beta coefficients were used to assess physician's age, duration of work in primary care and patient load impact on job strain, job demands and decision latitude. Results differences at the p = 0.05 level were considered as statistically significant.

## Results

### Descriptive statistics

Of the 197 respondents, 162 (82.2%) GP's were female, and 35 (17.8%) male. This is very similar to whole GP population in Lithuania. The GP ages ranged from 31 to 66 years (mean 44.2 years, 95% CI 42.9 – 45.4). GP's were investigated in 3 age groups: < 44 yr – N = 90 (45.7%); 45–54 yr – N = 85 (43.1%); 55 and > – N = 22 (11.2%).

Regarding to our data in general Lithuanian GP's have high patient load and are at risk of stress, they have high job demands and low decision latitude (table 2).

**Table 2:** Descriptive analysis of measured characteristics

**Characteristics**	**Values**		
	
	Mean	SD	95% CI
**Age**	44.2	9.0	42.9–45.4
**Patient load**	23.8	6.7	22.8–24.7
**Duration of work in primary care**	17.6	10.0	16.2–19.0
**Job demands**	37.1	6.8	36.2–38.1
**Decision latitude**	23.5	6.5	22.6–24.4
**Psychosocial stress**	5.0	1.2	4.8–5.2

### Logistic regression

The logistic regression beta coefficients showed that job strain development and higher job demands could be predicted by the shorter duration of GP practice. Otherwise older age for GP's can predict lower job demands and higher decision latitude. We found that lower decision latitude can be predicted by high patient load (table 3).

**Table 3:** Predicting coefficients of psychosocial stress, job demands and decision latitude

**Predictor**	**Psychosocial stress**		**Psychological workload demands**		**Job decisions latitude**	
	
	Beta	p-value	Beta	p-value	Beta	p-value
Age	0.009	0.13	**0.008**	**0.05**	**-0.008**	**0.01**
Duration of work in primary care	**-0.012**	**0.03**	**-0.009**	**0.02**	0.004	0,14
Patient load	-0.003	0.40	0.003	0.21	**-0.003**	**0.05**

In figures the interactions are graphically presented according to the method described by Aiken [34] and recognized in psychological research [35]. In terms of interactions we analysed job strain, job demands and decision latitude with respect to age and patients load. Values of the predictor variables were chosen one standard deviation below and above the mean.

**Figure 1:**
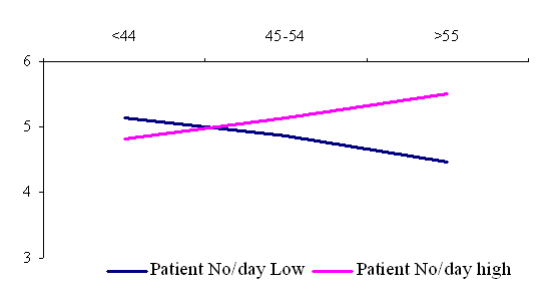
Interaction among general practitioner age and patient load in the prediction of job strain.

The interactions with regard to job strain (fig. 1) shows that GP's age and job strain are negatively associated to a low patient load. In other words, for older GP's job strain development have stronger associations with high patient load than young GP's.

**Figure 2:**
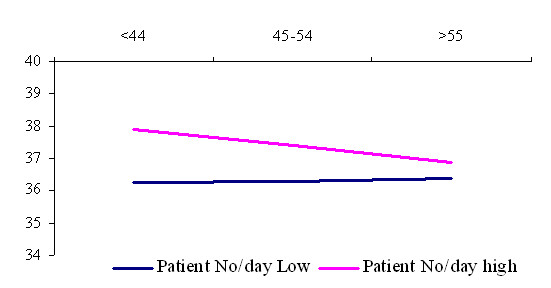
Interaction among general practitioner age and patient load in the prediction of job demands.

The age interactions with respect to psychological job demands (fig. 2) shows that job demands and GP age are slightly positively related at low numbers of patients per day. It shows that young GP's in terms of job demands more sensitively perceive increase in patient load that those in older age group.

**Figure 3:**
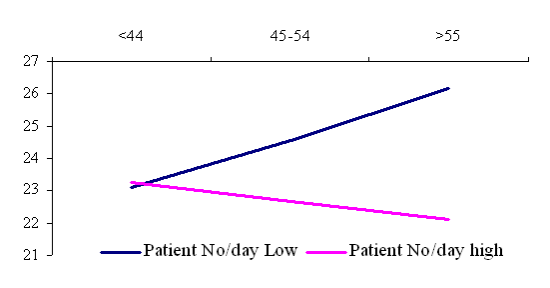
Interaction among general practitioner age and patient load in the prediction of decision latitude.

Regarding to job decision latitude (fig. 3), the interaction terms shows that higher decision latitude and older general practitioner's age are strongly related to a lower patient load, which means that these variables are positively but inversely associated with patient load. Decision latitude and patient load for younger GP's has week associations.

## Discussion

In the current social and political climate Lithuanian GPs face many stressors that are peculiar to the medical profession. However there are many stressors that are also attributed to the personality. GPs are the professionals who are at the forefront of helping patients to manage urgent health problems, and as gatekeepers they have to make decisions on patient's health; whether to send them to hospitals. Sometimes it can interfere with personal life that can cause negative feelings about work, frustration, tension and lack of time to make appropriate decisions [23].

Our study has highlighted a matrix of issues contributing to elevated levels of job strain. These issues are rarely attributable to a simple cause and effect formula but there are complex problems with the many linkages. Lithuanian GP's has indicated twofold age interaction with job strain because it depends on patient load. Work related stress development was hardly related to duration of working in primary care. GP's perceive higher job strain and higher job demands when they have shorter duration of GP practice. Older GP's are more vulnerable to job strain, when age interaction compared among low and high patient load groups. This also means different workload and job demands. It seems to be the confirmation of Cox definition of work related stress, where the concept includes an external demand and an internal perception that the response to the demand is uncomfortable: "Work related stress is a person's recognition of his/her inability to cope with demands relating to work, and his/her subsequent experience of discomfort" [34]. We found differences in perceived job demands and in objectively measured workload units. It can be explained within growing psychological adaptation to working environment with increasing duration of GP practice. We can see the same in fig 2. younger GP's are more vulnerable in perception of the increase in workload.

Peterson's substantial review found that detrimental work environments had social and psychological consequences for all [35]. He mentioned that the extent of decision-making power, decisions latitude, as well as overwork is related to job strain development. We can say more, namely that higher patients load can be a predictor of lower decision latitude and it seems also to be related to GP age. Our results highlighted that high patient load can cause decrease in decision latitude for the older age GP's and has only week associations to younger GP's.

Several weaknesses of the present study have to be mentioned. As main weakness of our study we see its cross-sectional nature, which precludes an evaluation of temporal precedence and causality of the observed associations. Karasek Job Strain model guided our hypothesis about causal relationships between age, patient load and work characteristics, explored causal relations should be interpreted carefully and longitudinal studies should be carried out in the future research.

Another limitation is the Karasek's Job Content Questionnaire it self. It was designed to be broadly applicable to a wide range of occupations. However, this generalisability inevitably means that factors that are specific to particular occupations may be overlooked. For example, job demands as it has been conceptualized and operationalised in this survey would not take into account some emotional demands that could be source of stress to general practitioners such as dealing with difficult patients or caring for the dying patients [35,36].

Third limitation is our exclusive reliance on self-reported rating scales, which raises the issue of systematic positive or negative response tendencies. Furthermore, as no scale is perfectly reliable, the associations between self-reported measures and self-reported workload appear to be weaker than they could be in reality. Several authors have argued that this phenomenon is not a major threat if interactions has been found [7,37].

On the positive side, our results were obtained among a sample of people working in general practice. Respondents were with similar education level that can be seen as strength of the investigation. The sample was sufficient regarding to sample size calculation and allow exploration of tendencies. The participation rate was acceptable, and the scales we used were previously validated instruments that retained their psychometric properties in our population [26]. Otherwise it is important to mention that generalisability of Karasek's model allow to us comparisons among different medical and non medical occupational groups and this is important factor selecting job strain model. One of the principal outputs of this article is a categorization of the characteristics into a series of domains, in order to provide consistent information on the prediction of job strain, job demands and decision latitude perception. Findings from this research have hopefully emphasized the importance of examining changes and associations between work characteristics and job strain among GP's before health care reform in Lithuania will be definitely implemented.

## Conclusions

Lithuanian GP's have high patient load and are at risk of stress, they have high job demands and low decision latitude. Job strain development and higher job demands can be influenced by shorter duration of general practice. Older GP's perceive less strain, lower job demands and higher decision latitude in case of low patient load. Young GP's decision latitude has week association to patient load. Regarding to changes in patient load younger GP's perceive it more sensitively as changes in job demands.

## Competing interests

The author(s) declare that they have no competing interests.

## Authors' contributions

GV designed the study, abstracted data, made data analysis, drafted and revised the manuscript.

SBA participated in initial study design, participated in data analysis and revised the manuscript.

All authors read and approved the final manuscript.

## Pre-publication history

The pre-publication history for this paper can be accessed here:


